# Mesenchymal Stem Cells Adaptively Respond to Environmental Cues Thereby Improving Granulation Tissue Formation and Wound Healing

**DOI:** 10.3389/fcell.2020.00697

**Published:** 2020-07-29

**Authors:** Dongsheng Jiang, Karin Scharffetter-Kochanek

**Affiliations:** ^1^Comprehensive Pneumology Center, Institute of Lung Biology and Disease, Helmholtz Zentrum München, Munich, Germany; ^2^Department of Dermatology and Allergic Diseases, Ulm University, Ulm, Germany

**Keywords:** mesenchymal stem cells, environment sensing, wound healing, granulation tissue, scarring, myofibroblasts, proliferation, inflammation

## Abstract

Granulation tissue formation constitutes a key step during wound healing of the skin and other organs. Granulation tissue concomitantly initiates regenerative M2 macrophages polarization, fibroblast proliferation, myofibroblast differentiation with subsequent contraction of the wound, new vessel formation, and matrix deposition. Impaired granulation tissue formation either leads to delayed wound healing or excessive scar formation, conditions with high morbidity and mortality. Accumulating evidence has demonstrated that mesenchymal stem cell (MSC)-based therapy is a promising strategy to ameliorate defects in granulation tissue formation and to successfully treat non-healing chronic wounds. In this review we give an updated overview of how therapeutically administered MSCs ensure a balanced granulation tissue formation, and furthermore discuss the cellular and molecular mechanisms underlying the adaptive responses of MSCs to cue in their direct neighborhood. Improved understanding of the interplay between the exogenous MSCs and their niche in granulation tissue will foster the development of MSC-based therapies tailored for difficult-to-treat non-healing wounds.

## Introduction

Cutaneous wound healing is characterized by three distinct yet overlapping phases including inflammation, proliferation, and remodeling (Singer and Clark, 1999; Wlaschek and Scharffetter-Kochanek, 2005; Eming et al., 2007). Granulation tissue formation is the hallmark of the proliferation phase. During granulation tissue formation, the polarization of reparative M2 macrophages, proliferation and differentiation of contractile myofibroblast, wound contraction, new vessel formation, and matrix deposition are concomitantly initiated (Gabbiani et al., 1971; Flanagan, 1998; Gabbiani, 1998; Hinz and Gabbiani, 2003; Hinz, 2007, 2016; Hinz et al., 2012; Jiang and Rinkevich, 2020). Recent studies revealed that myofibroblasts in granulation tissue derived from Engrailed-1 (En1)-positive lineage by lineage tracing studies (Rinkevich et al., 2015; Jiang et al., 2018) or from a subset that expresses delta like non-canonical Notch ligand 1 (Dlk-1) as the surface marker (Driskell et al., 2013). The initial cells and extracellular matrix proteins in the granulation tissue are mainly contributed by the subcutaneous fascia that underneath the dermal layer (Correa-Gallegos et al., 2019). Aberrations in granulation tissue formation result in non-healing chronic wounds or excessive scarring (Wynn and Ramalingam, 2012; Hu et al., 2018), both constitute major concerns for global healthcare.

Mesenchymal stem cells (MSCs) are multipotent progenitor cells residing in different tissues including skin (Hocking and Gibran, 2010; Jiang and Scharffetter-Kochanek, 2015). Apart from self-renewal and their differentiation capacity into several lineages, their paracrine impact on distinct resident and recruited cells is essential for tissue homeostasis and wound repair. Their relationship to En1 lineage positive fibroblasts or Dlk-1 positive fibroblasts is currently unresolved. Employing single cell sequencing analysis combined with *in vivo* tracing analysis and genetic deletion of quiescence or other markers have recently allowed to better define the origin and the lineage hierarchy of mesenchymal progenitors and stem cells in tissue and organ regeneration (Scott et al., 2019; Soliman et al., 2020).

MSCs have emerged as a potentially promising therapy for wound healing disorders of the skin and other organs. So far, adult tissue-derived MSCs have not entered clinical routine as fully approved products for the treatment of non-healing wounds, likely due to the unresolved hurdles with homogeneous isolation, and reliable and reproducible potency (Galipeau and Sensebe, 2018; Martin et al., 2019). However, there is ample evidence that MSCs enhance wound healing in preclinical wound models when applied on acute wounds (Li et al., 2006; Jiang et al., 2013), and more importantly to chronic wounds (Falanga et al., 2007; Javazon et al., 2007; Dash et al., 2009; Akita et al., 2010; Jimenez et al., 2012). MSCs promote granulation tissue formation, angiogenesis, and re-epithelialization, leading to accelerated wound closure. Moreover, MSCs establish a regenerative rather than fibrotic microenvironment, resulting in improved healing quality (Jiang and Scharffetter-Kochanek, 2015).

Accumulating evidence suggests that MSCs are endowed with the capacity for environmental sensing during granulation tissue formation and are able to mount adaptive responses, a superior property compared to conventional chemical-based or recombinant growth factor-based therapies (Basu et al., 2018; Vander Beken et al., 2019; Jiang et al., 2020b; Munir et al., 2020). This adaptive property allows for refinement of MSC-based therapies in near future. The application of MSCs at high numbers into wounds is currently the gold standard over manipulation of MSCs in their endogenous niche. In fact, endogenous MSCs are either depleted in chronic wounds or are themselves part of the pathogenic event (Lim et al., 2019) and, in consequence, cannot contribute to proper healing. In this review, we give recent cellular and molecular insights into how therapeutically applied MSCs behave as an environment sensing adaptive drugstore in granulation tissues during skin wound healing.

## Functional Roles of MSCs in Regulating Granulation Tissue Formation

### MSCs Improve Fibroblast Proliferation and Myofibroblast Activation

Impaired fibroblast proliferation leads to deficient granulation tissue formation. Fibroblast senescence, insufficient transforming growth factor beta 1 (TGF-β1) and platelet-derived growth factor (PDGF) signaling is known to result in defects of granulation tissue formation, which substantially delays the onset of proliferation phase. This is consistently observed in aging-related chronic wounds (Jiang et al., 2020a), diabetic foot ulcers (Falanga, 2005), pressure ulcer (Stanley et al., 2005), and chronic venous ulcers (Hasan et al., 1997; Agren et al., 1999).

The initial upsurge of MSC research was focused on their differentiation and transdifferentiation potential to replace the damaged tissue. Sasaki and colleagues reported that bone marrow-derived MSCs (BM-MSCs) are recruited into the wounds and contribute to wound repair by transdifferentiation into keratinocytes, endothelial cells and pericytes (Sasaki et al., 2008). Later on, accumulating evidence suggest that the engraftment efficiency and integration rate of exogenous MSCs in the repaired wounds are low. The contribution of MSCs to granulation tissue is predominantly through their trophic, paracrine and immunomodulatory functions by secretion of growth factors, proangiogenic factors, cytokines and chemokines, such as TGF-β, insulin-like growth factor-1 (IGF-1), prostaglandin E2 (PGE2), platelet-derived growth factor (PDGF), epidermal growth factor (EGF), hepatocyte growth factor (HGF), basic fibroblast growth factor (FGF-2), vascular endothelial growth factor (VEGF), angiopoietin-1, stromal cell-derived factor-1 (SDF-1), erythropoietin, monocyte chemoattractant protein-1 (MCP-1). More details are reviewed in Murphy et al. (2013); Jiang and Scharffetter-Kochanek (2015); Hu et al. (2018).

MSCs transplanted into full-thickness wounds augment proliferation of fibroblasts, and activate the fibroblast-to-myofibroblast transition. In physiological wounds, adipose tissue-derived MSCs (AT-MSCs) delivered by a medical-grade silicone carrier coated by plasma polymerization with a thin layer of acrylic acid (ppAAc) accelerate wound healing by promoting granulation tissue formation featured with higher numbers of alpha-smooth muscle actin (α-SMA)^+^ myofibroblasts and CD31^+^ vessel endothelial cells (Jiang et al., 2013). The ppAAc coating preserves the cellular properties of MSCs grown on the carrier and possesses the high delivery efficiency of MSCs when the carrier is applied topically to the wounds (Jiang et al., 2013).

Pathological conditions with defective granulation tissue formation often lead to chronic wounds. Patients with leukocyte adhesion syndrome type I (LAD-1), which is due to mutations in the *CD18* gene encoding the common β chain of β2 integrins, typically show impaired wound healing with reduced wound contraction and, in consequence, life-threatening infections (Anderson and Smith, 2001). The follow-up study with CD18-deficient (CD18^–/–^) mice, closely mimicking human LAD-1, revealed that delayed healing of full-thickness excisional wounds was due to severely impaired formation of granulation tissue and impaired wound contraction (Scharffetter-Kochanek et al., 1998). In this model, CD18^–/–^ neutrophils cannot bind to endothelial cells, thus cannot extravasate from vessels into the wound site. In addition, CD18^–/–^ neutrophils are resistant to apoptosis (Coxon et al., 1996; Weinmann et al., 2003). Therefore, the number of apoptotic neutrophils at the wound site was dramatically reduced. In addition, due to the loss of β2 integrins (CD18 deficiency), macrophages are unable to adhere to and subsequently phagocytose apoptotic neutrophils. Lacking this stimulus, the wound macrophages are incapable of secreting TGF-β1 (Peters et al., 2005). In consequence, the myofibroblast activation was substantially dysregulated with significantly reduced expression of the myofibroblast markers α-SMA and fibronectin in CD18^–/–^ wounds (Peters et al., 2005). Recently, we have demonstrated that the xenotransplantation of human AT-MSCs to CD18^–/–^ wounds are able to restore the low TGF-β1 concentration in the wound microenvironment of CD18^–/–^ mice. Through the release of human TGF-β1, transplanted MSCs promotes the proliferation and myofibroblast differentiation from resident mouse fibroblasts in wounds, profoundly enhances wound contraction and vessel formation, rescues granulation tissue formation, and accelerates wound healing in the CD18^–/–^ model (Jiang et al., 2020b). Several other frequently occurring chronic wounds are affected by a persistent inflammation phase and an almost complete lack of granulation tissue formation. Some of them will be discussed in more detail in section “MSCs Function as Guardians of Inflammation During Granulation Tissue Formation.”

### MSCs Reduce Contraction and Improve the Quality of Wound Healing

By contrast to conditions with reduced wound contraction, “over-healing” with hyperproliferation of fibroblasts and/or persistence of myofibroblasts in the granulation tissue cause contracture, fibrosis, and scarring frequently occurring in hypertrophic scars and keloid scars (Wynn and Ramalingam, 2012; Ud-Din and Bayat, 2020). MSC-based therapy can ameliorate fibrosis and promote tissue regeneration through suppressing wound contraction and tension (Uysal et al., 2014), reducing the number of myofibroblasts at the end of the proliferation stage (Stoff et al., 2009), and increasing wound tensile strength (McFarlin et al., 2006).

TNF-stimulated gene-6 (TSG-6) released from BM-MSCs is responsible for the reduction of granulation tissue formation at late stage of wound healing. MSC-derived TSG-6 suppresses the release of TNF-α from activated macrophage and concomitantly induces a switch from a high to an anti-fibrotic low TGF-β1/TGF-β3 ratio, which results in lower expression of α-SMA and less collagen deposition in the granulation tissue. Therapeutically administered MSCs also influence the collagen fiber arrangement in the restoration tissue of healed wounds. Compared to densely packed and disorganized thick collagen fibers in control scars, collagen fibers in MSC-injected scars are thinner and organized in a basket-weave pattern, which significantly enhance the tensile strength of the healed wounds (Qi et al., 2014). Human BM-MSCs engraftment inhibited the hypertrophic scarring in a rabbit ear hypertrophic scar model, through increased secretion of TSG-6 from MSCs when they undergo apoptosis (Liu et al., 2014). Interestingly, polymorphisms in the *TSG-6* gene enhance susceptibility to hypertrophic scar and keloid formation. Also, TSG-6 expression is significantly reduced in hypertrophic scars and keloids (Tan et al., 2011). Prostaglandin E2 (PGE2) has been suggested to be another anti-scarring molecule that upregulated in transplanted BM-MSCs in wounds (Nemeth et al., 2009). It reprograms T cell and macrophage responses, and subsequently induces IL-10 release and prevents excessive collagen deposition (Liechty et al., 2000). In addition, nitric oxide produced by MSCs in the wound can scavenge ROS and may prevent tissue fibrosis (Ferrini et al., 2002; Jackson et al., 2012). Of note, a recent study reveals that grafting AT-MSCs enriched fat reduces radiation-induced skin fibrosis and joint contracture, also reduces skin stiffness, increases skin elasticity, and decreases the proportions of pro-fibrotic CD26^+^Dlk1^+^ fibroblasts in the irradiated skin (Borrelli et al., 2020).

### MSCs Function as Guardians of Inflammation During Granulation Tissue Formation

Prolonged inflammation in wounds profoundly hinders granulation tissue formation and delays wound healing. For example, the defect in the transition of pro-inflammatory M1 to anti-inflammatory M2 leads to persistent release of TNF-α and hydroxyl radical from over-activated M1 macrophages. This persistent oxidative attack induces senescence of wound fibroblasts and causes non-healing or chronic wounds, often occurs in venous leg ulcers, peripheral occlusive arterial, pressure and diabetic ulcers (Mirza and Koh, 2011; Sindrilaru et al., 2011; Mirza et al., 2013; Krzyszczyk et al., 2018). Senescent fibroblasts adopt a senescence associated secretory phenotype (SASP) with persistent release of pro-inflammatory cytokines, chemokines and matrix-degrading metalloproteinases, among them TNF-α, IL-1 and IL-6, which in consequence, fuel a vicious cycle of M1 macrophage activation further delaying the switch to M2 macrophages and granulation tissue formation (Mirza and Koh, 2011; Sindrilaru et al., 2011).

The anti-inflammatory property enforces the most prominent beneficial effects of MSC-based therapies, which has been discussed in detail in a few excellent reviews (Prockop and Oh, 2012; Wang et al., 2016; Regulski, 2017; Munir et al., 2018). Despite the initial lung entrapment, exogenously administered MSCs migrate along cytokine gradients toward inflamed tissue and home to sites of injury, where they suppress inflammation in their local environments (Rustad and Gurtner, 2012). Therefore, they are ideally suited for the therapy of chronic wounds which are characterized by unrestrained and prolonged inflammation.

In physiological (acute) wounds, MSCs suppress the TNF-α-dependent inflammation and thereby increase anti-inflammatory M2 macrophage numbers (Jiang et al., 2013), through MSC-derived TSG-6 (Qi et al., 2014). Moreover, injection of dermal MSCs expressing ATP binding cassette subfamily B member 5 (ABCB5) into an iron overload chronic wound model in mice, closely mimicking chronic venous leg ulcers in patients, the release of interleukin-1 receptor antagonist (IL-1RA) from ABCB5^+^ MSCs was observed to fundamentally dampen inflammation and to shift the prevalence of unrestrained pro-inflammatory M1 macrophages toward repair promoting anti-inflammatory M2 macrophages at the wound site (Vander Beken et al., 2019). The good manufacturing practices (GMP)-grade ABCB5^+^ MSCs fulfill the regulatory requirements to be employed for the treatment of chronic wounds in humans (Tappenbeck et al., 2019). Of note, clinical phase IIa studies have recently been initiated (EudraCT numbers: 2015-000399-81, 2017-000233-31, and 000234-57) with promising results of the first studied patients suffering from CVU.

MSCs also ameliorate unrestrained neutrophil activation as reported in a mouse model of vasculitis. This beneficial MSC effect is enforced by augmenting intercellular adhesion molecule-1 (ICAM-1) on macrophages to engulf apoptotic neutrophils and, furthermore, by detoxifying the reactive oxygen species (ROS)-rich environment through enhanced release of soluble extracellular superoxide dismutase (SOD3) from injected AT-MSCs (Jiang et al., 2016, 2017a, b).

### MSCs Support Immune Response to Clear Infections

An interesting phenomenon has been observed during the MSC-based trials that under immunosuppressive regimens, the infection rates are not increased (Regulski, 2017). In line with that, cumulative data indicate that the immunoregulatory function of MSCs reveals high plasticity. In contrast to the strong immunosuppressive effect of MSCs on allogenic responses and persistent sterile inflammation, MSCs do not appear to suppress immune cell functionality in the presence of infectious agents, but rather facilitate the clearance of infections and accelerate wound repair.

The immunoregulatory fate of MSCs can be switched in accordance with the dynamics of inflammation, which depends on the strength of activation of the immune system, the types of inflammatory cytokines present (Wang et al., 2014). *In vitro*, the plasticity of mouse BM-MSC-mediated immunomodulation in response to fluctuations in inflammation levels has been demonstrated by the titration of IFN-γ and TNF-α (Li et al., 2012). Moreover, antigen-pulsed human BM-MSCs stimulated with a low dose of IFN-γ act as antigen-presenting cells and can thus activate antigen-specific cytotoxic CD8^+^ T cells (Chan et al., 2006). During viral infection, human BM-MSCs do not inhibit cytotoxic T lymphocyte (CTL) functions, but rather facilitate CTL responses by releasing interferon-gamma (IFN-γ) (Kang et al., 2005). In mice suffering from chronic *Staphylococcus aureus* infection with biofilm formation, therapeutically administered human BM-MSCs, when combined with antibiotics, significantly reduce bacterial numbers at the wound site and improve wound healing. The beneficial MSC effects on wound healing is due to the secretion of antimicrobial peptides and triggering the bactericidal functions of neutrophils such as phagocytosis and neutrophil extracellular trap (NET) formation (Chow et al., 2020). Recent clinical trials indicate that autologous transplantation of MSCs could vastly improve outcomes for patients suffering from multidrug resistant strains of *Mycobacterium tuberculosis* (Skrahin et al., 2014, 2016). MSC-based therapy has also demonstrated beneficial effects against parasite infections such as Malaria (Souza et al., 2015) and *Schistosoma japonicum* (Xu et al., 2012).

**FIGURE 1:**
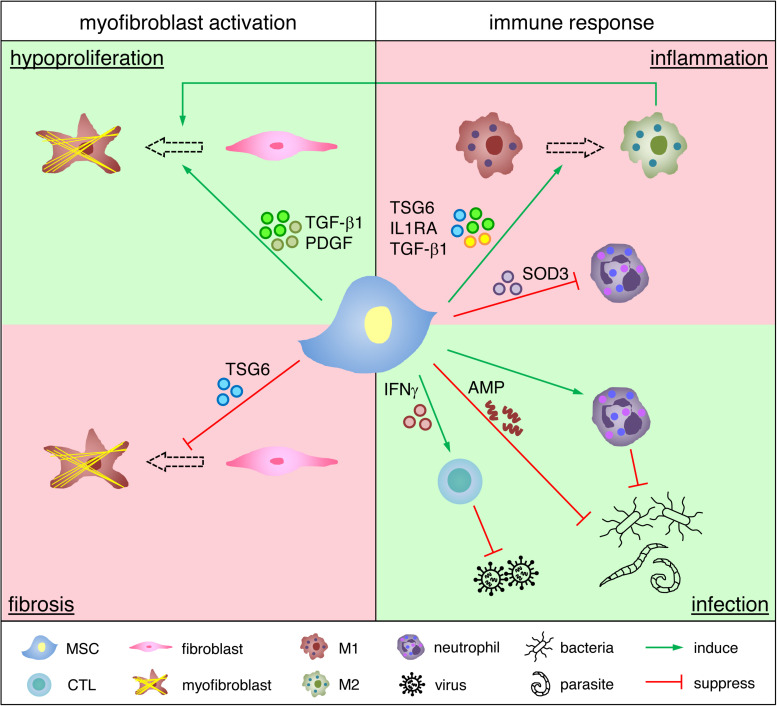
Functional roles of MSCs in regulating granulation tissue formation. MSCs promote fibroblast proliferation and myofibroblast differentiation in a hypoproliferative environment, whereas they suppress myofibroblast activation and reduce myofibroblast numbers in a fibrotic environment. MSCs induce M1 to M2 transition and inhibit neutrophil activation in an over-inflamed environment, whereas they support cytotoxic T lymphocytes (CTL) and neutrophils for the clearance of infectious agents. Due to space limitation only selected examples of how MSCs impact on granulation tissue are depicted.

A simplified scheme elucidating the modulatory roles of MSCs on myofibroblast activation and immune response during granulation tissue formation is summarized in Figure 1.

## Cellular and Molecular Mechanisms Underlying the Adaptive Responses of MSCs

**FIGURE 2:**
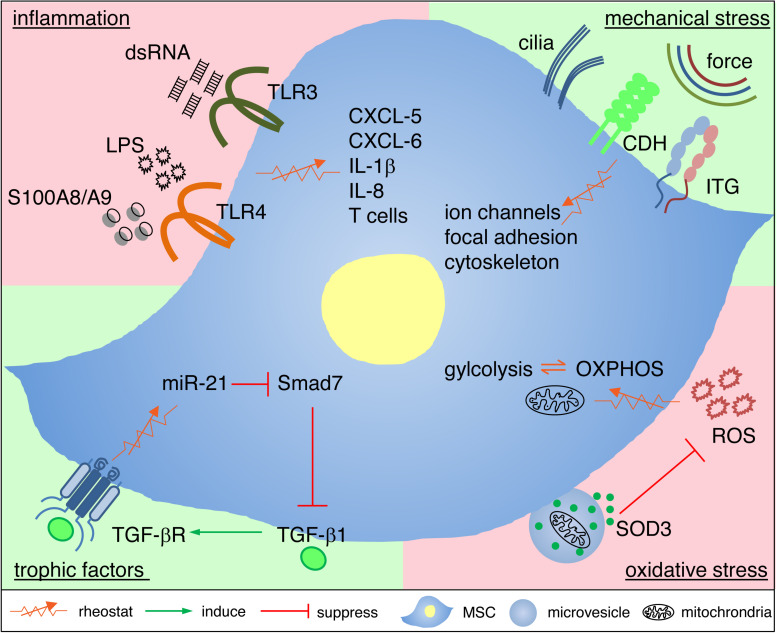
Cellular and molecular mechanisms underlying adaptive responses of MSCs. MSCs utilize rheostatic (sensing) mechanisms to respond to the local environment in granulation tissue with deficient or excessive trophic factors, inflammatory mediators, oxidative stress or mechanical stress. This adaptive response to cues from the microenvironment depends on distinct intracellular signaling, changes of the transcriptome and metabolic reprogramming. Adaptive MSC responses, in consequence, rebalance the partly hostile microenvironment, and thus protect themselves by rebalancing their niche, and this favors a shift to healing and tissue homeostasis. For further details see main text. CDH, cadherins; ITG, integrins; OXPHOS, oxidative phosphorylation, ROS, reactive oxygen species.

It is fascinating that MSCs can adaptively restore and attenuate defects in granulation tissue formation independent of whether myofibroblast proliferation is pathologically reduced or enhanced. Similarly, MSCs adaptively regulate the immune response depending on the requirements of the tissue and the organism. The molecular mechanisms underlying these seemingly reciprocal responses of MSCs have started to be unraveled in the recent decade. As to the question why MSCs have evolved sensing mechanisms and the ability to raise distinct adaptive responses to changing environmental cues is currently not fully understood. An interesting possibility could be that stem cells with regenerative potential and self-renewal capacity to maintain and recover tissue homeostasis would profit from protecting their blueprint, the DNA. Controlling noxious oxidative attacks, inflammation and infection by sensing and shaping their neighborhood would very much support DNA protection. Here we give an overview of how MSCs adaptively respond to trophic factors, inflammation, oxidative and mechanical stresses (Figure 2).

### MSCs Act as Rheostat of Trophic Factors to Re-establish Tissue Homeostasis

TGF-β signaling is the most important pathway that determines granulation tissue formation and wound healing outcome (Desmouliere et al., 1993; Margadant and Sonnenberg, 2010; Lichtman et al., 2016; Jiang and Rinkevich, 2020). Deficiency in TGF-β1 expression as in CD18^–/–^ (Peters et al., 2005) or dysregulated TGF-β1 signaling as in diabetic foot ulcers (Badr et al., 2012; Zhang et al., 2016) and chronic venous leg ulcers (Sindrilaru et al., 2011) result in chronic non-healing wounds, whereas excessive TGF-β1 leads to contracture and scarring as seen in hypertrophic scars and keloid scars (Jagadeesan and Bayat, 2007; Chalmers, 2011; Wynn and Ramalingam, 2012). Therefore, supplementation of not too much or too little TGF-β1 would be the preferred ideal situation. This, however, is technically difficult to achieve with recombinant TGF-β1 protein, in particular as the requirement for TGF-β1 dynamically changes during tissue repair and may not be the same in different wounds and different individuals. Recently, we have shown that human AT-MSCs are endowed with the unique sensing capacity for environmental TGF-β1 levels *in vitro* and *in vivo* at the wound site.

The sensing of actual TGF-β1 concentrations in the environment is initiated by binding of TGF-β1 to TGFβ receptors on MSCs. The activation of TGF-β receptors either suppress or upregulate the expression level of microRNA-21 (miR-21), depending on the TGF-β1 concentrations at the wound site. In murine LAD-1 wounds that represent an excellent model for chronic wounds with a profound TGF-β1 deficient state, activation of TGFβ receptors induces miR-21, which blocks the translation of Smad7, a negative regulator of TGF-β signaling. This, in consequence, promotes enhanced TGF-β1 release from MSCs. By contrast, overactivation of TGFβ receptors on MSCs at high TGF-β1 concentrations down-regulates miR-21 and, thus, no longer inhibits Smad7 translation, eventually suppressing the release of TGF-β1 from MSCs (Jiang et al., 2020b). The signaling circuit of TGF-β receptor – miR-21 – Smad7 – TGF-β1 adapts the release of MSC-derived TGF-β1 exactly to the demands at the wound site (Figure 2).

### Pattern Recognition and Danger Molecule Sensing

When a robust immune response is necessary to clear invading pathogens or to initiate repair responses, this is mainly initiated by a large amount of pathogen-associated molecular pattern (PAMP) and/or danger-associated molecular pattern (DAMP) at the wound site or in the endogenous environment of MSCs.

At the injury site, therapeutically applied MSCs have the capacity to mount an adaptive response by sensing PAMP via toll-like receptors (TLRs). The activation of TLR4 signaling in human AT-MSCs by the bacterial wall component lipopolysaccharide (LPS) increases the release of CXCL-5 (GCP2), CXCL-6 (ENA78), IL-1β and IL-8, and thereby augments the recruitment and activation of neutrophils and macrophages. Due to phagocytosis of increased numbers of apoptotic neutrophils, macrophages release more TGF-β1 and subsequently promote a strong myofibroblast-driven wound contraction and profoundly accelerate wound healing (Munir et al., 2020). In another study, Liotta and colleagues have shown that ligation of TLR3 and TLR4 on human BM-MSCs leads to the temporary inhibition of MSCs to suppress T cell proliferation. This is achieved by downregulation of the Notch ligand Jagged-1 expressed on the surface of MSCs, and thus blocking MSC’s suppressive instructions to the interacting T cells through their Notch receptors (Liotta et al., 2008). The authors hypothesize that this is an effective mechanism to block the immunosuppressive activity of MSCs and therefore to restore an efficient T-cell response in the course of dangerous infections sustained by double-stranded RNA viruses or Gram-negative bacteria. Of note, activation of TLRs do not affect the immunophenotype or differentiation potential of MSCs (Liotta et al., 2008).

Although S100A8/A9 (MRP8/14), a DAMP and an endogenous TLR4 ligand, primed AT-MSCs also lead to accelerate wound healing, the adaptive response of MSCs to S100A8/A9 is quite different when compared to that to LPS priming (Basu et al., 2018). Changes in the transcriptome of LPS primed MSCs enhance mainly the numbers and the activity of neutrophils, and thereafter in the second instance macrophages are activated following engulfment of apoptotic neutrophils with enhanced release of TGF-β1 (Munir et al., 2020). By contrast, in S100A8/A9 primed MSCs mainly a transcriptome responsible for “the big clean up” of tissue debris at the wound site was observed with increased expression of MSCs genes which upon release of the encoded proteins enhance the phagocytotic capacity of macrophages. In addition, genes enhancing niche protection and energy homeostasis most likely play a major role in accelerating wound healing after injection of S100A8/A9 primed MSCs (Basu et al., 2018). It would be interesting to tease out how MSCs are able to mount distinct adaptive responses in their transcriptome through TLR4 in a ligand-specific manner.

TLR signaling in MSCs is regulated by various miRNAs (Abdi et al., 2018). For example, high miR-21 expression can enhance TLR signaling by its transcriptional suppression of Wnt (Fafian-Labora et al., 2017), whereas let-7b miRNA suppresses TLR signaling by directly targeting TLR4 (Ti et al., 2015). The interplay between TLR pathway and miRNAs adds further complexity and flexibility to TLR signaling of licensed MSCs in response to their changing environment.

### Adaptive Metabolic and Antioxidant Responses

In their native environment, MSCs are in a quiescent state characterized by low proliferation and high multi-potentiality. In this state, MSC metabolism is primarily maintained by glycolysis (Katajisto et al., 2015). Upon wounding, rapid proliferation for tissue repair and the demand for enhanced ATP production in MSCs depends significantly more on oxidative phosphorylation (Pattappa et al., 2011). This metabolic shift, likely mediated through hypoxia-inducible factor (Ma et al., 2009), regulates the global MSC secretome and exosome biosynthesis (Yuan et al., 2019), and thereby can tightly control MSCs and their paracrine response to environmental cues at the injury site.

Impaired mitochondrial function has been associated with chronic wounds featured by persistent inflammation and impaired granulation tissue formation (Peppa et al., 2009). Particularly, in diabetic foot ulcers, fibroblasts and endothelial cells bearing damaged mitochondria are prone to apoptosis, which further impairs the cellularity of granulation tissue, angiogenesis and perfusion of the newly forming restoration tissue with oxygen and nutrients (Berlanga-Acosta et al., 2013). MSCs are able to respond to such mitochondria malfunction under stress or injury by transferring mitochondria to damaged and stressed cells at the wound site (Murray and Krasnodembskaya, 2019). The mitochondria transfer between MSCs and target cells can be uni-directional (Court et al., 2020) or bi-directional (Figeac et al., 2014) and occurs via tunneling nanotubes or microvesicles, depending on local environmental cues.

It was first reported in 2006 that mitochondria and mitochondrial DNA (mtDNA) can be actively transferred from human BM-MSCs to ethidium bromide pretreated recipient cells with mutated and depleted mtDNA, to rescue the aerobic respiration in recipient cells (Spees et al., 2006). The *in vivo* evidence that the transfer of intact mitochondria can contribute to tissue repair was provided several years later. BM-MSCs attached to LPS-injured mouse alveolar epithelial cells in a connexin-43 gap junction-dependent manner, and transfer intact mitochondria by forming nanotubes and microvesicles. The mitochondrial transfer resulted in increased alveolar ATP concentrations and contribute to tissue repair (Islam et al., 2012). Under oxidative stress, MSCs employ the arrestin domain-containing protein 1 (ARRDC1)-mediated microvesicles for transfer of healthy mitochondria to macrophage. This mitochondria transfer results in enhanced bioenergetics in macrophages, while decreasing intracellular oxidative stress in MSCs (Phinney et al., 2015). Similarly, in response to an inflammatory environment, human MSCs from varying sources including bone marrow, menstrual blood and umbilical cord, have been shown to transfer mitochondria to T cells. Surprisingly, it results in increased glycolysis in T cells and induce differentiation and activation of CD25^+^FoxP3^+^ regulatory T cells (Court et al., 2020). Such adaptive responses of MSCs restrict inflammation, reduces tissue damage and expedites the repair machinery.

Another mechanism of an adaptive MSC response to protect against elevated oxidative stress in the local environment is the release of SOD3, a soluble form of superoxide dismutase, which detoxifies superoxide anion radicals in their niche. Therapeutically injected AT-MSCs utilize this mechanism to suppress unrestrained neutrophil activation and to alleviate severe tissue damage in a murine immune-complex mediated vasculitis model. In consequence, AT-MSCs reduce the superoxide anion concentrations in the microenvironment and consequently prevented enhanced neutrophil death, neutrophil extracellular trap formation and spillage of matrix degrading neutrophil elastase, gelatinase and myeloperoxidase (Jiang et al., 2016).

### Sensing Mechanical Stress

MSCs sense mechanical stimuli such as tissue stiffness, compression, tension and hydrostatic pressure. These parameters are often changed in granulation tissue during wound healing and are abnormal in pathological scars and contractures (Delaine-Smith and Reilly, 2012; Steward and Kelly, 2015).

The adhesion of MSCs to matrix and other cells are key for the mechanotransduction of MSCs. Therefore, integrins responsible for cell-matrix binding, and cadherins responsible for cell-cell binding, are hypothesized to function as mechanosensors. For instance, β3 integrin mediates myogenic differentiation of BM-MSCs on soft matrix (Yu et al., 2013), whereas α2 integrin enforces osteogenic differentiation on stiff substrate (Shih et al., 2011). Cadherin-2 is mandatory for myogenesis of MSCs (Gao et al., 2010), and Cadherin-11 is involved in establishing a profibrotic niche with active TGF-β (Lodyga et al., 2019).

The engagement of integrins and cadherins triggers downstream events including ion channel opening for Ca^2+^ influx (Mizuno, 2005; Liedert et al., 2008), focal adhesion assembly with focal adhesion kinase (FAK) activation (McBeath et al., 2004) and cytoskeleton remodeling (Lv et al., 2015), to promote different cellular responses and MSC differentiation. Recently, microtubule-based organelles called primary cilia extending dynamically from human BM-MSCs have been observed. They serve as “multifunctional antenna” that sense both chemical and mechanical signals (Hoey et al., 2012a, b).

MSCs not only respond to mechanical cues, but can also acquire a memory of stiffness of the local environment, even 2 weeks after the initial stimulation. The memory of stiffness is mediated by miR-21, under the control of myocardin-related transcription factor-A (MRTF-A) (Li et al., 2017). This is interesting as miR-21 is also required for sensing and responding to environmental TGF-β1 (Jiang et al., 2020b), and TGF-β1 regulates collagen synthesis and tissue contraction both contributing to stiffness.

It is worthy to note that the current knowledge of molecular details of the mechanosensing is mainly derived from *in vitro* systems with 2D or 3D MSC cultures. It is known that 2D culture especially with stiff plastic surface drastically changes the cellular properties of MSCs, including morphology, migration mechanism, differentiation capacity, gene expression profile and subsequent secretome. Engineered 3D culture systems that eliminate apical-basal polarization while still paying attention to both cellular access of soluble nutrients and physical constraints on cell morphology and proliferation are closer to the mechano-environment of the *in vivo* condition (Smith et al., 2017; Zhou et al., 2017; Doolin et al., 2020). However, *in vivo* situations are far more complex and clinically relevant given the unresolved fibroproliferative conditions of keloid and scar formation. Therefore, *in vivo* studies which further advance our understanding how MSCs sense and integrate downstream signals from multiple mechanosensors, and how MSCs subsequently respond to shape their surrounding matrix will be of major interest for future investigations.

## A Disrupted MSC Niche Leads to Loss of Regenerative Potential and Promotes Scarring

Tissue resident MSCs are mainly found at perivascular locations in different organs including skin (Crisan et al., 2008; Dulauroy et al., 2012; Yamanishi et al., 2012; Lemos and Duffield, 2018; Vander Beken et al., 2019). This location allows MSCs to rapidly detect local cues within the vessels and adjacent tissue, including the occurrence of PAMPs and DAMPs or the reduction in O_2_ concentrations. Recently, it became evident that if the endogenous tissue resident MSCs is forced to leave their homeostatic niche, or in case they cannot adequately deal and adapt to the changing environment, they become a source of myofibroblasts in fibrotic diseases (El Agha et al., 2017; Lemos and Duffield, 2018). This is a very important and clinically relevant notion which imposes high responsibility on medical doctors to consider the original niche of stem cells before transplanting them in a therapeutic intent into a non-fitting environment. In fact, inconsiderate injection of AT-MSCs into the eye to treat aging-related macular degeneration, the major cause of blindness in the elderly, resulted in enhanced formation of myofibroblast. Due to their contractile properties, the myofibroblasts tear off the retina with a sudden and result in vision loss (Kuriyan et al., 2017).

Two very recent studies have identified a subpopulation of perivascular MSCs in the heart and muscle, which are characterized by the expression of PDGFRα, stem cell antigen-1 (Sca-1, Ly6A) and the tumor repressor hypermethylated in cancer 1 (Hic1) (Scott et al., 2019; Soliman et al., 2020). Both studies have shown that Hic1^+^ MSCs play important roles in the homeostasis and repair of heart and skeletal muscle, differentiate into different cell types including fibroblasts and are kept in a resting state by Hic1 (Kim and Braun, 2020). The transcription repressor Hic1 is required for maintaining quiescence, since deletion of Hic1 leads to activation and expansion of MSCs, and subsequently leads to pathological fibrosis of heart (Soliman et al., 2020) and skeleton muscle (Scott et al., 2019), respectively. The exact molecular targets of Hic1 remain elusive.

In skin, dermal ADAM12^+^ MSCs, or Dlk1^+^ MSCs at a preferred perivascular niche are responsible for the maintenance of tissue turnover and homeostasis (Desmouliere et al., 1993; Driskell et al., 2013). However, during skin wounding, in a hypoxic and myofibroblast-inductive microenvironment, with high level of platelet-derived serotonin (Dees et al., 2011), Th2 cytokines IL4/IL-13 (Kodera et al., 2002), or connective tissue growth factor (CTGF, CCN2) (Lee et al., 2010), these endogenous dermal MSCs subpopulation may contribute to skin fibrosis and scarring through activation of 5-hydroxytryptamine receptor 2B (5-HT2B), IL-4 receptor alpha (IL-4Rα) (Nguyen et al., 2020), and TGF-β1 (Lipson et al., 2012) pathways, respectively.

Endogenous MSCs in an environment where they are incapable to detoxify enhanced oxidative stress become damaged themselves and likely lose their reparative capacity. For instance, MSCs isolated from patient with atherosclerosis have impaired mitochondrial function, and lost their immunosuppressive function against T cell proliferation (Kizilay Mancini et al., 2018). MSCs reprogram their metabolic activity from glycolysis during tissue homeostasis to oxidative phosphorylation during wound repair, resulting in accumulation of cytotoxic metabolic byproducts. This adaptive alteration reduces the metabolic fitness of endogenous MSCs, and decreases the basal autophagy and mitophagy rate, and subsequently leads to higher rate of cellular senescence and reduced regenerative potency (Ho et al., 2017; Singh et al., 2017).

Taking the consideration of the fact that perivascular dermal MSCs comprise only about 0.3–2.5% of total mesenchymal cells in the skin (Chen et al., 2007; Haniffa et al., 2007; Vander Beken et al., 2019), the number of endogenous MSCs is not sufficient to shape the hostile environment. However, when administered at the wound site or into tissue at high numbers, the adaptive responses of MSCs are able to restore the niche to a balanced state to facilitate high quality wound repair and possibly skin regeneration.

## Modifying Scaffold to Simulate Wound Environment to Potentiate MSCs Efficacy

MSC delivery using polymer-based biomaterials as scaffold has been the subject of intense investigation. Scaffold materials may be of natural, synthetic, or composite origin and engineered using a multitude of approaches including porogen leaching, electrospinning, molecular self-assembly, and phase separation. Scaffold can provide mechanical support and act as a niche for MSCs to improve their survival and paracrine activity that eventually promote angiogenesis, reepithelialization and wound healing. The advantages of using scaffold for MSC-based therapy has been nicely summarized in the recent reviews (Yildirimer et al., 2012; Dash et al., 2018; Hu et al., 2018). For example, the AT-MSC-seeded elastin scaffold demonstrated rapid cell proliferation with deposition of new ECM. Experimentation with the seeded scaffold *in vivo* with a murine excisional wound model revealed greater would closure (Machula et al., 2014).

On the other hand, the seeded MSCs have shown to adapt and modify the material property of scaffolds, such as porosity, surface topographies, and stiffness. For example, AT-MSCs on the electrospun fibers produced significantly higher levels of anti-inflammatory and pro-angiogenic cytokines compared to those cultured on microplates (Su et al., 2017). The authors concluded that the topography of fibrous scaffolds can provide unique microenvironment to modulate the paracrine function of AT-MSCs. The underlying molecular mechanism is not clear yet, although upregulated NFκB signaling in MSCs has been demonstrated.

Current scaffold research focus on the enhancement of MSC survival, proliferation and differentiation. However, that may not be exclusively essential for the beneficial effects on wound healing. In the authors’ opinion, the secretome released from MSCs continuously adapting to microenvironmental changes is key to facilitate reparative or regenerative processes needed for tissue homeostasis. Therefore, modification of scaffolds may be developed to simulate the microenvironment of the target tissue, which may induce the adaptive responses of the seeded MSCs. Such pre-conditioning of MSCs may potentiate their therapeutic efficacy. Some attempts have moved toward this direction, for example, modifications to collagen scaffolds enriched with biomacromolecules such as glycosaminoglycans (GAGs) or laminin or hyaluronic acid have been generated to replicate the wound ECM environment (Liu et al., 2008; Catanzano et al., 2015). More detailed bioactive cues mimicking disease-specific niche such as changes in fluid composition, mechanical stress, cell density, and nutrient levels could be considered for scaffold design, aim to not only promote wound repair but also scarless regeneration.

## Conclusion and Future Perspectives

The therapeutic potential of MSC-based therapy for chronic wounds is largely dependent on the ability of MSCs to release soluble factors and in a paracrine manner modulate granulation tissue formation rather than to engraft and differentiate/transdifferentiate into host tissue. Regardless of the caveats in their identity or source, MSCs have been reported to have beneficial effects on both accelerating wound healing and reducing scarring. The advantage of an MSC-based therapy over a recombinant growth factor cocktail-based therapy relies on the unique capacity of MSCs to sense and re-establish a reparative and regenerative local environment in response to changing requests of the wound.

The studies we have discussed here collectively indicate that MSCs are licensed to exert their reparative capacity after stimulation with environmental cues, such as PAMP and/or DAMP through TLRs, or growth factors and/or inflammatory mediators through respective receptors. This licensing-to-execution mode of responses makes MSCs highly suited to be therapeutically exploited as “smart adaptive drugstore” that perfectly meets to the specific and changing demands of defined wounds.

The here discussed original studies focus on MSCs from a particular tissue source, commonly BM-MSCs, AT-MSCs or skin connective tissue derived MSCs; however, MSCs are present in almost all types of mesenchymal tissues. It is an open question whether MSCs of different tissue origins all possess environmental adaptation capacity, or MSCs from one particular source are supreme in eliciting adaptive response, therefore better suited for clinical application. In addition, whether allogenic MSCs are as good as autologous MSCs in adaptive responses remain elusive. Moreover, we need to keep in mind that the essential role of MSCs in pathology of chronic wound healing disorders is not attributed to MSCs alone but is unique and directly linked to the disease-specific microenvironment. Therefore, the adaptive response of MSCs cannot be assumed as a general phenomenon, but require careful validation for distinct MSCs populations and, importantly, in a disease-specific manner.

Although current data clearly demonstrate the adaptive responses of therapeutically administered MSCs in wound healing, such roles of endogenous MSCs have yet to be investigated, due to the lack of specific markers to trace and monitor MSCs *in vivo*. It is certain that research into the mystery of tissue-resident MSCs will bring new insight into the roles of these unique cells in physiological and various pathophysiological conditions. The new knowledge we acquire from the endogenous licensing signals would feedback to optimize the preparation protocols of MSCs tailored for the clinical treatments of chronic wounds and even to shift the healing outcome from scarring toward skin regeneration.

## Author Contributions

DJ and KS-K wrote the manuscript and prepared the figures. Both authors contributed to the article and approved the submitted version.

## Conflict of Interest

The authors declare that the research was conducted in the absence of any commercial or financial relationships that could be construed as a potential conflict of interest.
